# High serum miR-223-3p expression level predicts complete response and prolonged overall survival in multiple myeloma patients undergoing autologous hematopoietic stem cell transplantation

**DOI:** 10.3389/fonc.2023.1250355

**Published:** 2023-09-27

**Authors:** Damian Mikulski, Mateusz Nowicki, Izabela Dróźdż, Małgorzata Misiewicz, Kacper Piotr Kościelny, Karol Okoński, Kinga Krawiec, Ewelina Perdas, Agnieszka Wierzbowska, Wojciech Fendler

**Affiliations:** ^1^ Department of Biostatistics and Translational Medicine, Medical University of Lodz, Lodz, Poland; ^2^ Department of Hematooncology, Copernicus Memorial Hospital in Lodz, Lodz, Poland; ^3^ Department of Hematology and Transplantology, Copernicus Memorial Hospital in Lodz, Lodz, Poland; ^4^ Department of Hematology, Medical University of Lodz, Lodz, Poland; ^5^ Department of Clinical Genetics, Medical University of Lodz, Lodz, Poland

**Keywords:** aHSCT, autologous hematopoietic stem cell transplantation, complete response, CR, MiR-223-3p, miRNA, multiple myeloma

## Abstract

**Introduction:**

AHSCT is the treatment of choice for newly diagnosed patients with transplant-eligible multiple myeloma (MM). However, considerable variability in response to autologous hematopoietic stem cell transplantation (AHSCT) results in only 50% of patients achieving complete response (CR) after AHSCT, which is directly associated with improved progression-free and overall survival (OS). In this study, we aimed to investigate the potential predictive role of selected serum miRNAs in MM patients who underwent AHSCT.

**Patients and methods:**

Serum expression level of 6 miRNAs: miR-221-3p, miR-15b-5p, miR-223-3p, miR-320c, miR-361-3p, and miR-150-5p was evaluated in 51 patients who underwent AHSCT. Blood samples were collected at two time points: before conditioning chemotherapy (T1) and fourteen days after transplant (+14) (T2).

**Results:**

All selected miRNAs significantly changed their expression level across the procedure- two were up-regulated after AHSCT: hsa-miR-320c (FC 1.42, p<0.0001) and hsa-miR-361-3p (FC 1.35, p=0.0168); four were down-regulated: hsa-miR-15b-5p (FC 0.53, p<0.0001), hsa-miR-221-3p (FC 0.78, p=0.0004), hsa-miR-223-3p (FC 0.74, p=0.0015) and hsa-miR-150-5p (FC 0.75, p=0.0080). Notably, before AHSCT, hsa-miR-223-3p was down-regulated in International Staging System (ISS) III patients (FC=0.76, p=0.0155), and hsa-miR-320c was up-regulated (FC=1.27, p=0.0470). These differences became non-significant after AHSCT. Eight (15.69%) patients achieved CR before AHSCT and 17 patients (33.33%) at +100 days after AHSCT. In multivariate logistic regression analysis, achievement of CR after induction and hsa-miR-223-3p at T1 were independent predictors of CR after AHSCT. In multivariate Cox regression analysis, hsa-miR-223-3p at T1 expression level was associated with prolonged OS (HR 0.06, 95%CI: 0.00 - 0.99, p=0.0488).

**Conclusion:**

Serum expression of has-miR-223-3p is a predictor of CR and prolonged OS in MM patients undergoing AHSCT.

## Introduction

Multiple myeloma (MM) is a hematologic malignancy characterized by the clonal proliferation of plasma cells in the bone marrow, producing aberrant monoclonal protein. In 2018, the global incidence of MM was 160,000 cases, and the global myeloma mortality rate was 106,000 patients (1). In recent years, a significant breakthrough was made in patient outcomes due to novel medications such as proteasome inhibitors (PI), immunomodulatory drugs (IMiDs), and monoclonal antibodies (2, 3). Despite the advances in treatment options, MM remains incurable, and almost all patients eventually relapse.

High-dose melphalan (HDM), followed by autologous hematopoietic stem cell transplantation (AHSCT) was introduced in the 1980s and significantly increased the survival of MM patients (4, 5). In 2019, more than 28.700 AHSCT procedures were performed in Europe, and the majority (55%) were performed in MM patients (6). The significance of AHSCT in treating MM patients has been questioned as the availability of novel targeted therapies increases (7, 8), leading to several randomized trials to establish the role of AHSCT in the novel therapy era (9–11). Despite concerns, HDM-AHSCT remains the treatment of choice for newly diagnosed, transplant-eligible MM patients (12).

However, there is still considerable variability in response to AHSCT. Some patients achieve long-term remission, while up to 19% of patients experience early relapse (ER) within 18 months after transplant (13). CR after AHSCT was previously shown to be associated with superior OS in MM patients (14). On the other hand, early relapse after AHSCT is strongly associated with reduced survival, independently from cytogenetic risk (13, 15). Identifying predictive biomarkers that might stratify patients depending on their likelihood of achieving response following AHSCT is therefore essential to direct maintenance and or/consolidation treatment and monitoring protocols and improve patient outcomes.

An emergent class of biomarkers – circulating miRNAs has shown tremendous promise in oncology (16). MiRNAs are small non-coding RNAs that play a crucial role in post-transcriptional gene regulation by binding to the 3’ untranslated region (3’UTR) of target mRNAs, leading to their degradation or translational repression (17). MiRNAs have been linked to various cellular processes, including cell proliferation, apoptosis, and differentiation, and their dysregulation has been associated with the development of cancer non- and cancer diseases (18–20). MiRNAs have been implicated as essential actors in a variety of cardiovascular disorders, neurodegenerative disorders, and immune dysfunctions (21–23). In addition, miRNAs have been shown to regulate a myriad of physiological processes including cardiac remodeling and vascular homeostasis, as well as synaptic plasticity and neuronal survival mechanisms (24, 25). MiRNAs found in serum and exosomes have exhibited utility as valuable biomarkers for diagnostic and prognostic purposes, while also offering insights into treatment refractoriness and potential therapeutic interventions (26–28). In recent years, miRNAs have emerged as potential factors associated with MM pathogenesis, including tumor initiation and progression; they can also be used for MM diagnosis, risk stratification, and drug response prediction (29–34).

In this study, we aimed to investigate the potential prognostic role of selected serum miRNAs in MM patients who underwent AHSCT (35). We hypothesized that these miRNAs may be associated with critical biological processes involved in MM progression and predict the patient’s response to treatment.

## Materials and methods

### Patients and treatment

The study group consisted of prospectively recruited patients treated with AHSCT at the Department of Hematology and Transplantology, Copernicus Memorial Hospital in Lodz, Poland. The inclusion criteria were (1) age >18 years; (2) diagnosis of MM; (3) qualification to AHSCT during the treatment schedule. The exclusion criteria included contraindications to AHSCT, mainly Hematopoietic Cell Transplantation-Comorbidity Index score (HCT-CI) ≥ 3. The myeloablative conditioning regimen was high-dose melphalan (200 mg/m2) (12). All patients have received granulocyte-colony-stimulating factor (G-CSF) from +4 day after AHSCT (72 h after transplantation) until engraftment. Engraftment was defined as the first three days with a neutrophil count >0.5 x10^9^. Response to treatment and relapse/progression were classified based on International Myeloma Working Group (IMWG) criteria (36, 37). Cytogenetic abnormalities were classified according to the IMWG classification (38–40). The study was conducted according to good clinical and laboratory practice rules and the principles of the Declaration of Helsinki. All procedures were approved by the local ethical committee (The Ethical Committee of the Medical University of Lodz, No RNN/424/19/KE). Each patient signed the informed consent for all examinations and procedures.

### miRNAs

In this study, we selected for investigation miRNAs that were previously reported to be deregulated in biology of MM, including tumor growth, drug resistance, and immune modulation: hsa-miR-15b-5p (41), hsa-miR-320c (42), hsa-miR-221-3p (43), hsa-miR-223-3p (44), hsa-miR-361-3p (45), hsa-miR-150-5p (46). In contrast to previous studies, we chose to concentrate on miRNAs expression in serum due to the clinical availability, reliability, and simplicity of introducing blood-based biomarker tests into clinical practice.

### Samples collection

Blood samples were collected at two time points: before conditioning chemotherapy (T1) and fourteen days after transplant (+14) (T2). Blood samples were drawn into anticoagulant-free containers and kept at room temperature for 30 minutes. Serum was separated by centrifugation at 1500 g for 15 minutes at 4°C. The serum samples were kept at -80°C until analysis.

### RT-qPCR

The expression levels of selected miRNAs were quantified by RT-qPCR using the miRCURY LNA miRNA Custom PCR Panels (QIAGEN) using the Roche Lightcycler 480 qPCR platform. Total RNA, including miRNA, was extracted using the miRNeasy Serum/Plasma Advanced Kit (QIAGEN), following a phenol-free protocol, in accordance with the manufacturer’s instructions. Briefly, a 200 µl volume of serum was mixed with Buffer RPL to facilitate effective lysis condition and to stabilize RNA. Subsequently, the resulting mixture was combined with Buffer RPP and subjected to centrifugation to separate out contaminants like plasma proteins. Isopropanol was then introduced to the supernatant, creating favorable conditions for RNA molecules exceeding 18 nucleotides in length to adhere to a silica membrane. The kit employs a spin-column technology-based procedure. The sample, treated in this manner, was then applied to the RNeasy UCP MinElute spin column. During this step, RNA binds to the membrane, while undesired analytes and contaminants are removed through subsequent washing stages. In the concluding phase, total RNA molecules exceeding 18 nucleotides in length were eluted from the column using 20 µL of RNase-free water and stored at −20°C until further use.

The expression levels of selected miRNAs were quantified by RT-qPCR using the miRCURY LNA miRNA Custom PCR Panels (QIAGEN). Briefly, 2 µL of cDNA was synthesized from the obtained total RNA including mature miRNAs (<200 bp), using the miRCURY LNA Reverse Transcription Kit (QIAGEN) according to the manual provided by the manufacturer. Mature miRNAs were polyadenylated by poly(A) polymerase and reverse transcribed into cDNA using oligo-dT primers. The cDNA was stored at −20°C until further analysis. Next, a premix of 3 µL of cDNA template (diluted 1:30), 5 µL of 2X miRCURY LNA SYBR Green PCR Master Mix and filled with RNase-free water to the final volume of 10 µL, was aliquoted into the PCR plate. Real-time PCR was performed on a LightCycler480 II Real-Time PCR System (Roche, Pleasanton, CA, USA). The reaction was performed at 95°C for 2 min, followed by 55 amplification cycles at 95°C for 10 s and 56°C for 1 min. Absolute quantification of miRNA was determined using the LightCycler® 480 Software, Version 1.5 (Roche, Mannheim, Germany).

### Statistical analysis

The expression of miRNAs was calculated according to the ΔCt method (47). RT-qPCR data were normalized by using the mean expression value of two miRNAs in a given sample (hsa-miR-27b-3p and hsa-miR-148b-3p) which were found to be the most stable factor according to NormiRazor Software in our previous study on AHSCT recipients (35, 48). The formula used to calculate the normalized Ct values was: Normalized ΔCt = (mean Ct of hsa-miR-27b-3p and hsa-miR-148b-3p) − Ct miRNA of interest.

This approach results in higher values for higher miRNA expression enabling straightforward biomarker interpretation.

Differential expression analysis was performed using an unequal variances t-test. Paired measurements were compared using paired t-test. Differences between groups for continuous variables were compared using the Student’s t-test or U Mann-Whitney test depending on the variable distribution. Logistic regression univariate and multivariate were conducted to establish the association of selected miRNA and clinical variables with CR achievement after AHSCT. WEKA was used to train a classifier using as an outcome of CR achievement after AHSCT. Cox univariate and multivariate models were performed to establish the prognostic value of miRNAs. Kaplan-Meier plots and log-rank tests were performed to visualize and compare survival curves.

Our study was conducted according to the pre-planned sample size estimation, as we expected a difference in the experimental and control means of 0.5 ΔCt with a standard deviation of 0.5, we needed to study 17 experimental subjects and 17 control subjects to be able to reject the null hypothesis that the means of the experimental and control groups are equal with probability (power) 0.8 and type I error probability of 0.05.

All statistical analyses were conducted using Statistica Version 13.1 (TIBCO, Palo Alto, CA, USA) and R programming language (version 4.0.2). Normalized miRNA expression data with crucial variables were provided in **Supplementary File 1**. Most of the analyses utilized OmicSelector R package (49). P values lower than 0.05 were considered statistically significant.

## Results

### Study group characteristics

The study group consisted of 27 women and 24 men with a mean age of 59.5 ± 8.9 (range 35.5-71.9) years. Among CRAB symptoms at the time of diagnosis, the most prevalent symptoms were bone disease (66.7%) and anemia (19.6%). In the majority of patients (72.6%) IgG was the dominant paraprotein, followed by light chain disease (LCD) (17.6%). Nineteen patients (37.3%) had stage III disease, according to the International Staging System (ISS). Among 13 patients with available cytogenetic assessment, 4 had high-risk cytogenetic abnormalities.

Most patients (68.6%) received a bortezomib-based induction regimen, most commonly VCD (bortezomib, cyclophosphamide, dexamethasone, 56.9%). Seventeen patients (33.3%) had refractory disease to the first line and required subsequent systemic therapy line before AHSCT- most commonly in second-line patients received bortezomib-based regimens (29.4%) with or without thalidomide (VTD/VCD/VD) and lenalidomide-dexamethasone (Rd) (23.5%). Two patients underwent tandem AHSCT; samples were collected during the first procedure. Eight patients achieved complete response (CR) before AHSCT. The detailed characteristics of the study cohort were provided in **Table 1**.

**Table 1:** Characteristics of the study group.

Characteristics	Total
Number of patients	51 (100)
Gender	M: 24 (47.06)
	F: 27 (52.94)
Age	59.53 ± 8.85
mean + SD (range)	(35.46–71.89)
Treatment	
VCD	29 (56.86)
VTD	5 (9.80)
CTD	12 (23.53)
Other	5 (9.80)
Bortezomib-based induction regimen	
Yes	35 (68.63)
No	16 (31.37)
Paraprotein	
IgG	37 (72.55)
IgA	4 (7.84)
IgM	1 (1.96)
LCD Lambda	5 (9.80)
LCD Kappa	4 (7.84)
Bone disease at diagnosis	34 (66.67)
Calcium > 2.75 mmol/L at diagnosis	4 (7.84)
HB < 10 g/dL at diagnosis	10 (19.61)
Creatinine > 2 mg/dL at diagnosis	6 (11.76)
International Staging System (ISS)	I-18 (35.29)
	II-14 (27.45)
	III-19 (37.25)
Conditioning regimen	
Mel-175, 140, 100	17 (33.3)
Mel-200	34 (66.67)
Cytogenetics*	N = 13
t(4;14)	1
t(14;16)	2
del(17p)	1
t(11;14)	2
Cytogenetic risk groups	
High Risk	4 (7.84)
Standard Risk	9 (17.65)
Unknown Risk	38 (74.51)
Response before AHSCT	
CR	8 (15.69)
VGPR	22 (43.14)
PR	16 (31.37)
SD	3 (5.88)
missing	2 (3.92)
Response after AHSCT	
CR	17 (33.33)
VGPR	18 (35.29)
PR	9 (17.65)
SD	2 (3.92)
PD	1 (1.96)
missing	4 (7.84)

^*^Cytogenetics data were available for 13 patients (25.49%).

### The AHSCT course and outcome

Most patients (N=34, 66.7%) received a melphalan 200 mg/m2 conditioning regimen, 17 (33.3%) received a reduced dose of melphalan. Patients received a median of 3.9 x 10^6^ CD34+ cells/kg (IQR 3-5.3 x 10^6^ CD34+ cells/kg). The median ANC engraftment was 11 days (interquartile range, IQR: 11-12), and the median PLT engraftment time was 16 days (IQR: 13-19). At +100 days after AHSCT, 17 patients (33.33%) achieved CR, 18 patients (35.29%) had very good partial response (VGPR), 9 (17.65%) had a partial response (PR), two patients had stable disease and one patient had disease progression. There was no reimbursement for standard lenalidomide maintenance after AHSCT in Poland at the time of the study, so only five patients received maintenance treatment: 2 patients received lenalidomide, two patients received thalidomide, and one patient received bortezomib in monotherapy. The median PFS time in our study cohort was 29.83 months, 95% CI: 22.77 - 37.00 months, and the median OS was 58.83 months, 95% CI: 37.43 - 93.90 months.

### Influence of the AHSCT procedure and other clinical factors on the miRNA expression levels

**Figure 1:**
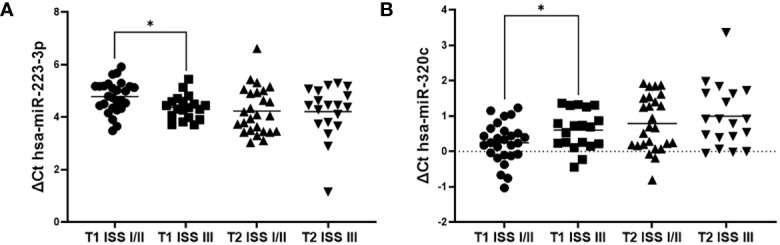
Comparison of miRNAs expression between ISS I/II and stage III at T1 and T2 study time points. **(A)** hsa-miR-223-3p; **(B)** hsa-miR-320c.

Overall, 3 of 102 (2.94%) samples were excluded due to technical issues from analysis. All selected miRNAs significantly changed their expression level across the procedure- two were up-regulated after AHSCT: hsa-miR-320c (FC 1.42, p<0.0001) and hsa-miR-361-3p (FC 1.35, p=0.0168); four were down-regulated: hsa-miR-15b-5p (FC 0.53, p<0.0001), hsa-miR-221-3p (FC 0.78, p=0.0004), hsa-miR-223-3p (FC 0.74, p=0.0015) and hsa-miR-150-5p (FC 0.75, p=0.0080) (**Table 2**). Notably, before AHSCT, hsa-miR-223-3p was down-regulated in ISS III patients (FC=0.76, p=0.0155) and hsa-miR-320c was up-regulated (FC=1.27, p=0.0470) (**Supplementary Table 1**). These differences became non-significant after AHSCT (FC 0.98, p=0.9249 and FC 1.15, p=0.4195, respectively) (**Figure 1**). There were no significant differences in miRNA expression level according to the myeloma type (LCD vs other), induction treatment (bortezomib-based regimen vs other), age (>60 years vs younger), presence of the CRAB symptoms at the time of diagnosis. There were also no significant differences in high-risk cytogenetic group compared to standard risk group, however, it should be noted that cytogenetic data were available only for 13 patients (25.49%). In addition, there were no significant differences in miRNAs expression in patients with and without CR before AHSCT.

**Table 2:** Expression of selected miRNAs before and after AHSCT.

miR	Before AHSCT Mean ΔCt	Before AHSCT SD	After AHSCT Mean ΔCt	After AHSCT SD	FC	log2FC	p-value
hsa-miR-15b-5p	0.23	0.76	-0.68	0.72	0.53	-0.91	<0.0001
hsa-miR-320c	0.40	0.56	0.90	0.84	1.42	0.50	<0.0001
hsa-miR-221-3p	0.46	0.51	0.09	0.59	0.78	-0.37	0.0004
hsa-miR-223-3p	4.62	0.60	4.18	0.95	0.74	-0.43	0.0015
hsa-miR-361-3p	-5.20	0.58	-4.77	1.08	1.35	0.43	0.0168
hsa-miR-150-5p	-0.34	0.78	-0.76	0.93	0.75	-0.42	0.0080

*The comparison was performed using paired t-test.

### Factors affecting response to AHSCT

**Figure 2:**
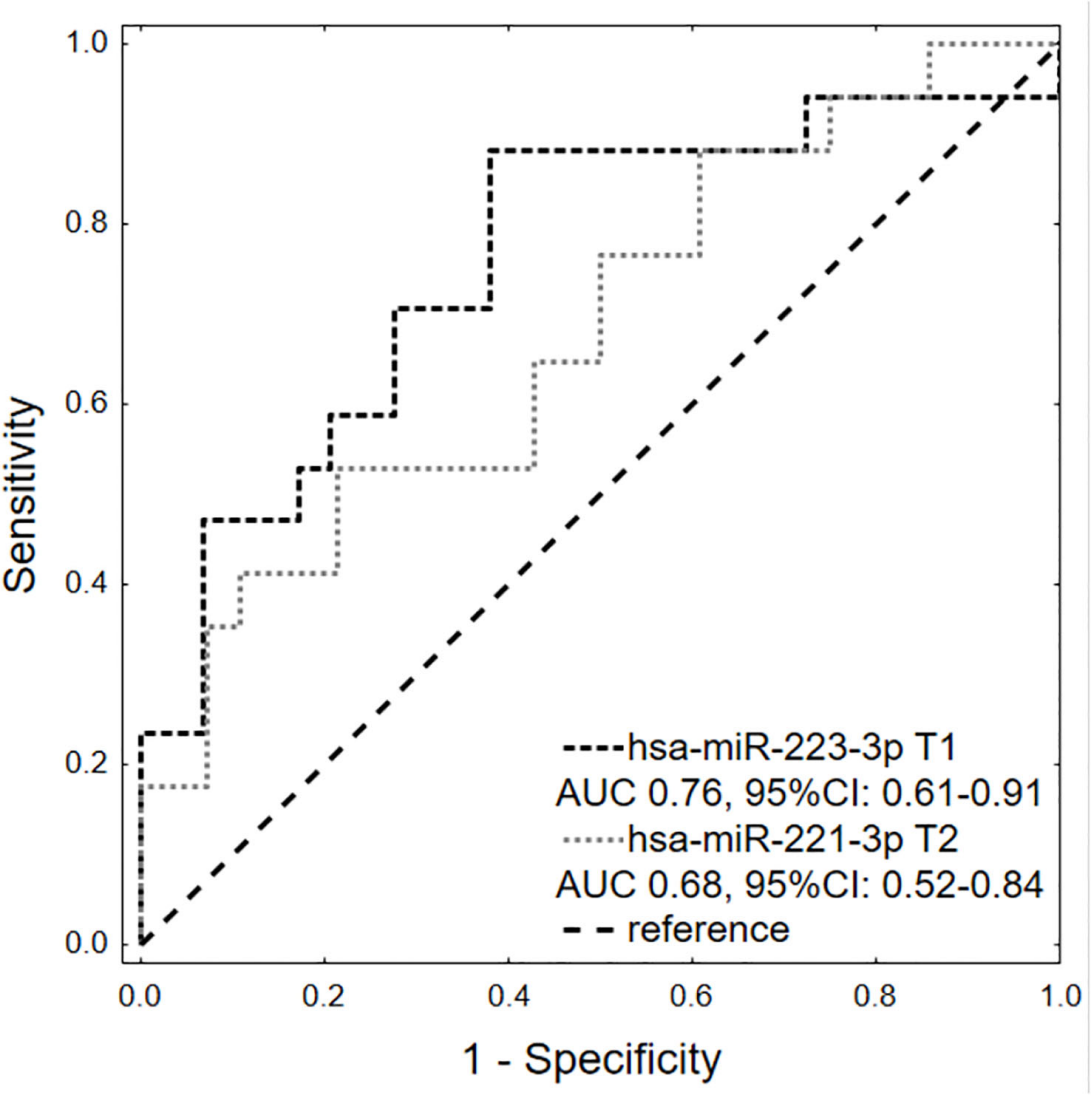
Comparison of hsa-miR-223-3p at T1 and hsa-miR-221-3p at T2 in predicting CR at +100 days after AHSCT.

To investigate the potential factors associated with CR achievement after AHSCT, univariate logistic regression of clinical variables and miRNAs expression levels were conducted. We found that CR achievement before AHSCT was the only significant clinical factor associated with complete response after AHSCT (OR 23.10, 95%CI: 2.53-210.89, p=0.0054) (**Table 3**). ISS III (OR 0.25) and ≥2 chemotherapy lines before AHSCT (OR 0.31) were associated with poorer response, however, they did not yield a statistically significant effect (p=0.0596 and p=0.1027, respectively). Among investigated miRNAs, two were significantly associated with CR after AHSCT: hsa-miR-223-3p at T1 (OR 5.76, 95%CI: 1.63-20.28, p=0.0065) and hsa-miR-221-3p at T2 (OR 3.57, 95%CI: 1.09-11.75, p=0.0362). hsa-miR-223-3p (AUC 0.76, 95%CI: 0.61-0.91) had generally higher discriminative power for differentiating CR from non-CR patients than hsa-miR-221-3p (AUC 0.68, 95%CI: 0.52-0.84), however, the difference was not statistically significant (p=0.4983). ROC analysis showed that hsa-miR-223-3p expression level T1 at a cut point of 4.494 achieved sensitivity and specificity of 88.2% and 62.1%, respectively (**Figure 2**). hsa-miR-221-3p T2 expression level had at a cut point of 0.374 reached 52.9% sensitivity and 78.6% specificity. In multivariate logistic regression analysis, only CR before AHSCT and hsa-miR-223-3p at T1 were independent predictors of CR after AHSCT.

**Table 3:** Univariate and multivariate logistic regression analyses of factors associated with CR achievement after AHSCT.

Variable		Univariate				Multivariate			
		OR	95%CI Lower	95%CI Upper	p	OR	95%CI Lower	95%CI Upper	p
CR before AHSCT		23.10	2.53	210.89	0.0054	38.55	3.04	489.24	0.0048
≥2 lines before AHSCT		0.31	0.07	1.27	0.1027				
Cytogenetic risk group	Unknown	*Reference*							
	Standard Risk	0.21	0.02	1.90	0.1662				
	High Risk	1.71	0.22	13.56	0.6094				
Maintenance treatment		0.65	0.06	6.72	0.7145				
Melphalan dose	Mel 200	0.60	0.18	2.01	0.4028				
	Reduced dose	*Reference*							
Induction regimen	Bortezomib-based	2.89	0.69	12.02	0.1448				
ISS	I/II	*Reference*							
	III	0.25	0.06	1.06	0.0596				
Age	<60	*Reference*							
	>60	0.49	0.15	1.63	0.2438				
Paraprotein type	Other	Reference							
	LCD	1.00	0.22	4.61	1.0000				
hsa-miR-221-3p T1		2.97	0.83	10.59	0.0926				
hsa-miR-15b-5p T1		0.78	0.36	1.70	0.5329				
hsa-miR-223-3p T1		5.76	1.63	20.28	0.0065	4.72	1.14	19.59	0.0327
hsa-miR-320c T1		0.73	0.26	2.10	0.5647				
hsa-miR-361-3p T1		0.82	0.29	2.27	0.6954				
hsa-miR-150-5p T1		1.37	0.63	2.98	0.4222				
hsa-miR-221-3p T2		3.57	1.09	11.75	0.0362	3.29	0.61	17.76	0.1659
hsa-miR-15b-5p T2		1.18	0.51	2.73	0.6936				
hsa-miR-223-3p T2		1.70	0.84	3.44	0.1390				
hsa-miR-320c T2		0.76	0.37	1.58	0.4628				
hsa-miR-361-3p T2		1.30	0.75	2.26	0.3432				
hsa-miR-150-5p T2		0.92	0.48	1.73	0.7903				

### Development of classification model of CR

To evaluate the discriminant power of hsa-miR-223-3p and clinical variables in discriminating patients with CR after AHSCT, a model was trained using CostSensitiveClassifier from the set of meta-classifiers of the WEKA software. CR before AHSCT and hsa-miR-223-3p expression at T1 were used as input variables. RandomTree classifier was used with weights adjustment applied during learning due to class imbalance (14 CR vs 32 non-CR cases). Our model in 10-fold cross-validation preserved its diagnostic potential with an accuracy of 84.78% (95%CI: 71.13%-93.66%), sensitivity- 78.57% (95%CI: 49.20% to 95.34%) and specificity- 87.50% (95%CI: 71.01% to 96.49%). WEKA dataset and model file are provided as **Supplementary Files 2**, **3**.

### The impact of miRNAs on OS in AHSCT recipients

**Figure 3:**
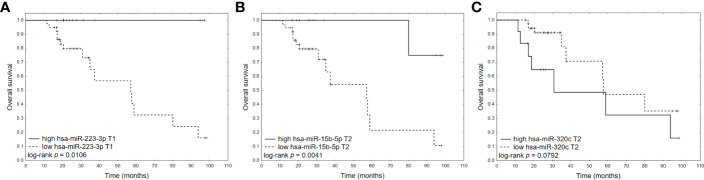
KM plots for miRNAs significant in univariate Cox analyses: **(A)** hsa-miR-223-3p at T1; **(B)** hsa-miR-15b-5p at T2; **(C)** hsa-miR-320c at T2. To compare survival probability, the miRNA ΔCt values were dichotomized into patient groups with lower and higher miRNA expression, defined as one upper quartile and three lower quartiles.

None of the analyzed clinical variables significantly affected OS in univariate Cox regression analyses (**Table 4**). Among selected miRNAs, hsa-miR-223-3p at T1(HR 0.37, 95%CI: 0.14- 0.97, p=0.0432), hsa-miR-15b-5p at T2 (HR 0.47, 95%CI: 0.22-1.00, p=0.0493) and hsa-miR-320c at T2 (HR 1.98, 95%CI: 1.04-3.74, p=0.0362) had significant impact on OS (**Figure 3**). In multivariate analysis, the two most significant clinical variables in univariate analyses with p<0.1- Hgb<10 at diagnosis and CR after AHSCT and established prognostic factor- ISS were included with significant miRNAs in univariate analysis. In this model, only hsa-miR-223-3p at T1 retained its significance and was an independent prognostic factor of OS.

**Table 4:** Univariate and multivariate Cox regression analysis of OS in AHSCT recipients.

Variable		Univariate				Multivariate			
		HR	95% CI Lower	95% CI Upper	P value	HR	95% CI Lower	95% CI Upper	P value
Age >60 years		2.55	0.72	8.98	0.1463				
Cytogenetic risk group	Standard	*Reference*							
	Unknown	0.48	0.12	1.86	0.3236				
	High Risk	0.90	0.09	8.83	0.8003				
Sex	F	1.40	0.50	3.88	0.5192				
Melphalan dose	Mel 200	*Reference*							
	Reduced dose	0.60	0.12	2.93	0.5303				
ISS	I/II	*Reference*				*Reference*			
	III	1.30	0.47	3.56	0.6151	0.28	0.05	1.77	0.1764
LCD		1.99	0.55	7.18	0.2956				
Calcium > 2.75 mmol/L at diagnosis		1.81	0.45	7.21	0.4024				
Creatinine > 2 mg/dL at diagnosis		0.59	0.13	2.77	0.5051				
HGB< 10 at diagnosis		0.13	0.01	1.28	0.0808	0.04	0.00	1.15	0.0606
Bone disease at diagnosis		0.92	0.28	2.99	0.8876				
Induction regimen	Bortezomib-based	0.47	0.15	1.51	0.2069				
≥2 lines before AHSCT		2.06	0.72	5.84	0.1762				
Maintenance treatment		1.70	0.48	6.08	0.4120				
CR before AHSCT		0.74	0.09	5.99	0.7817				
CR after AHSCT		0.15	0.02	1.12	0.0649	0.71	0.08	6.76	0.7695
hsa-miR-221-3p T1		0.57	0.22	1.42	0.2262				
hsa-miR-15b-5p T1		1.05	0.55	2.00	0.8775				
hsa-miR-223-3p T1		0.37	0.14	0.97	0.0432	0.06	0.00	0.99	0.0488
hsa-miR-320c T1		1.37	0.55	3.44	0.5039				
hsa-miR-361-3p T1		0.59	0.21	1.61	0.3008				
hsa-miR-150-5p T1		1.22	0.57	2.64	0.6049				
hsa-miR-221-3p T2		0.37	0.13	1.07	0.0667				
hsa-miR-15b-5p T2		0.47	0.22	1.00	0.0493	2.06	0.49	8.61	0.3223
hsa-miR-223-3p T2		0.57	0.31	1.06	0.0759				
hsa-miR-320c T2		1.98	1.04	3.74	0.0362	1.09	0.50	2.38	0.8190
hsa-miR-361-3p T2		1.02	0.69	1.52	0.9222				
hsa-miR-150-5p T2		0.99	0.54	1.82	0.9828				

## Discussion

This study identified the predictive and prognostic significance of hsa-miR-223-p in MM patients undergoing AHSCT. In our cohort, 17 patients (33.33%) had a CR at +100 days after AHSCT. In multivariate models, hsa-miR-223 was an independent factor of both achieving CR and for OS in multivariate survival analysis. Our research focused on miRNA expression in serum samples, which is a non-invasive and clinically feasible method for miRNA evaluation. Serum miRNAs are known to be relatively stable and easily identified and quantifiable, making them attractive as potential biomarkers. Our study uniquely evaluated miRNA expression in serum samples explicitly obtained during the AHSCT procedure, providing a more accurate assessment of their predictive role in this clinical setting. Furthermore, within the specific context of AHSCT, serum miRNAs have the potential to capture systemic changes and mirror the overall response of the entire organism, including the microenvironment of the bone marrow niche. This aspect is particularly crucial in the realm of MM biology concerning the AHSCT procedure. Released into circulation by various cell types, both hematopoietic and non-hematopoietic, serum miRNAs offer a more comprehensive insight into the prognosis of outcomes.

In this study, we selected miRNAs that have been previously reported to be dysregulated in the biology of MM, including tumor growth, drug resistance, and immune modulation. Recently, Papanota et al. showed that low expression of miR-223-3p in CD-138+ plasma cells in MM patients was related to inferior OS (44). In this study, 76 newly diagnosed MM patients were evaluated and only 28% of patients were subsequently referred to the AHSCT. On the other hand, in our study, using non-invasive methods, we could easily provide valuable insight of hsa-miR-223-3p on prognosis omitting bone marrow biopsy. MiR-223 is widely expressed in the hematopoietic system and serves as a crucial modulator of hematopoietic differentiation by orchestrating the expression of hematopoietic stem cells, erythroid cells, and granulocyte-monocyte progenitors at various phases of development (50). Wang et al. found that miR-223, together with other miRNAs- let-7a, miR-15a, miR-20a, miR-21, miR-106b, and miR-361 were decreased in the bone marrow microenvironment, peripheral blood, and CD138+ plasmocytes of MM (41). Moreover, it was hypothesized that down-regulation of these miRNAs might parallel disease progression from the precursor lesion of monoclonal gammopathy of unknown significance (MGUS) to the entirely malignant and symptomatic phase of myeloma. In addition, miR-223-3p was also reported to be downregulated in bortezomib-resistant MM cell lines and patients (51). In this study, the authors found that silencing of circular RNA chaperonin containing TCP1 subunit 3 (circ-CCT3) enhanced the sensitivity of bortezomib-resistant MM cells to bortezomib via modulating miR-223-3p. miR-223-3p increased bortezomib sensitivity by inhibiting bromodomain-containing 4 (BRD4). Taken together, we could hypothesize that hsa-miR-223-3p acts in a similar manner on the cellular and tissue level.

We also demonstrated that all 6 investigated miRNAs significantly changed their expression after AHSCT compared to baseline- two were up-regulated- hsa-miR-320c and hsa-miR-361-3p, and four were down-regulated- hsa-miR-15b-5p, hsa-miR-223-3p, hsa-miR-221-3p and hsa-miR-150-5p. Notably, measurement of miRNAs expression before and after AHSCT allowed us to demonstrate that baseline differences in hsa-miR-223-3p and hsa-miR-320c expression in regard to ISS stage rebalanced after AHSCT. It was demonstrated that after AHSCT, unique changes in the immunological system occur. For example, regulatory T cells (Tregs) decline after AHSCT with the cooccurring expansion of CD8^+^ T cells (52). Chung et al. described a subpopulation of exhausted/senescent CD8^+^ T cells, which were present in higher levels in patients who relapsed at +3 months after AHSCT but before clinically confirmed progression, indicating potential in discriminating patients in higher risk of relapse (52). In recent study by Parmar et al., comprehensive assessment of humoral and cellular tumor immune microenvironment (iTME) was performed (53). Two distinctive patterns of cellular iTME were described, one of them was associated with higher levels of naïve and terminally differentiated/exhausted T cells. This group had inferior outcomes in terms of time to progression and OS. The reason for that may be reduced immunosurveillance- naive T cells have not been yet activated, whereas differentiated effector T cells which lost their ability to control the disease. Notably, this effect was independent of other established high-risk factors, including cytogenetics. It is possible to speculate that other unknown immunological factors beyond T cells may contribute to the outcomes of AHSCT. The iTME is a complex ecosystem that involves interactions between various immune cells, tumor cells, and the tumor microenvironment. Serum miRNAs may act as signaling molecules that facilitate communication between components of iTME (54). They have the potential to regulate gene expression and influence cellular functions in recipient cells, contributing to the regulation of cell proliferation, differentiation, apoptosis, immune responses, and metabolism in recipient cells and tissues.

In our study, miR-320c was significantly up-regulated after AHSCT compared to the baseline level. In general, miR-320 family members were reported to be tumor suppressors in MM. miR-320a enhances apoptosis and inhibits tumor growth by targeting pre-B-cell leukemia transcription factor 3 (*PBX3*) (55). On the other hand, the oncogene *EZH2* was reported to inhibit the expression of miR-320c to promote MM development (56). In our study miR-320c expression increase in serum may demonstrate one of the potential mechanisms of action of AHSCT increasing suppressor miRNAs. On the other hand, according to the Human miRNA tissue atlas by Ludwig et al., the main tissues with miR-320c high expression are the skin and small intestine (duodenum). That is why the increase in serum expression may also result from common AHSCT complications- oral and intestinal mucositis (57).

We also noted the upregulation of hsa-miR-361-3p after AHSCT. Similarly, recently it was found that upregulation of miR-361-3p inhibited MM cells viability and enhanced apoptosis through targeting tumor necrosis factor receptor-associated factor 6 (TRAF6) (45). A microarray profiling of specimens and cell lines revealed that 1,799 miRNAs were found to be abnormally expressed in MM samples, with miR-361-3p being down-regulated (46). Multiple studies demonstrate that miR-361-3p plays a crucial role in cancer and metastasis. For example, Chen W. et al. discovered that miR-361-3p inhibits tumor growth in non-small-cell lung cancer and that its antitumor activity may be related to the inhibition of the target gene *SB2B1 (* 58). According to the Human miRNA tissue atlas main organs with high has-miR-361-3p are the thyroid, bones, and spleen (57). In summary, the direct cause and mechanism of the increase of serum has-miR-361-3p in MM patients after AHSCT are not clear; however, the potential suppressor role of this miRNA was reported.

In preclinical studies, melphalan sensitivity of MM cells was inversely proportional to miR-221/222 expression (43). In the presence or absence of human bone marrow stromal cells, inhibition of miR-221/222 overcame melphalan resistance and induced apoptosis of MM cells. Combining melphalan and miR-221/222 inhibition decreased MM proliferation with significant upregulation of the pro-apoptotic BBC3/PUMA protein. In our study, AHSCT caused a global decrease in the serum of MM patients.

Up to day, only several studies have evaluated the role of miRNAs in MM patients undergoing AHSCT (59–67). However, differences in study designs, including sample collection timing and miRNA evaluation methods, make direct comparisons of results challenging. Some studies evaluated miRNA expression before AHSCT (66) or at the time of diagnosis (61), some studies reported results with a mixed population of MM and refractory lymphoma patients (65, 67). However, the main limitation of these studies may be the reduced number of studied miRNAs limiting the biological impact of complex miRNA-mRNA interactions (65–67).

Among miRNAs evaluated as predictive factors for early relapse (ER) after AHSCT in MM patients was miR- 193a-5p (66). Park et al. proposed a multivariate model of 1-year PFS, where ISS II/III (HR 3.495, p=0.011) and higher expression of miR-193a-5p at pre-AHSCT (HR 0.286, p<0.001) were significant variables. In another study, Rafiee et al. demonstrated that both miR-125b in extracellular vesicles (HR 2.2) and in plasma (HR 6.7) at mobilization before AHSCT were independent factors of relapse (65). However, in this study, both MM and lymphoma patients were included. In a large cohort of 156 newly diagnosed MM patients, who received bortezomib and dexamethasone as induction and AHSCT, Manier et al. identified two exosomal miRNAs- let-7b and miR-18a, which lower expression were independent predictors of shorter both PFS and OS (61). However, as samples were collected at the diagnosis, these results seem to be rather prognostic for MM course rather than predictive for AHSCT outcome. Similarly, Navarro et al. evaluated serum miRNA expression at the time of diagnosis and at the time of CR)achievement after AHSCT (60). They found that patients at the time of CR with higher expression of miR-19b (6 vs. 1.8 years; *P*< 0.001) or miR-331 (8.6 vs. 2.9 years; *p* = 0.001) had longer PFS.

Several studies have assessed the role of miRNAs in relation to engraftment time. Rafiee et al. (67) evaluated four miRNAs expression, miR-125b, mir-126, miR-150, and miR-155 as potential engraftment predictors and the miR-155 expression after the administration of the conditioning regimen was the most significant predictor of platelet/neutrophil engraftment. However, in this study, among 50 included patients, only 24 were MM patients. In another study, the serum expression of miR-223-3p at day +7 of AHSCT was positively correlated with the engraftment time (62).

The study findings have several potential applications in the field of MM and miRNA biomarkers. The results indicate that the expression levels of miRNAs, especially hsa-miR-223-3p, could be used as biomarkers to predict the treatment response and outcomes of AHSCT patients and can contribute to the optimization of treatment. Especially, potentially patients with low has-miR-223-3p may be candidates for more intensive posttransplant treatment. Recently, more intensive posttransplant treatments, like carfilzomib, lenalidomide and dexamethasone (KRD) are being proposed (10, 68). Even more, ongoing randomized trials address whether CAR T-cell or other T-cell-engaging therapies could replace up-front AHSCT as a standard treatment. For example, CARTITUDE-6 is a phase 3 trial comparing cilta-cel and AHSCT in patients receiving initial induction therapy with daratumumab, bortezomib, lenalidomide and dexamethasone (DVRd) (69).

Our study has several limitations. Firstly, it should be mentioned that only two patients in our cohort received lenalidomide in maintenance treatment after AHSCT, as at the time of enrollment, there was no lenalidomide reimbursement in this indication in Poland (70). However, our cohort was mainly treated with bortezomib-based regimens, contrary to the previous studies on miRNAs in AHSCT recipients. Secondly, a significant limitation is missing cytogenetic data. In Poland, the availability of cytogenetic testing for multiple myeloma was limited in the previous several years. Even if obtained, cytogenetics had minimal impact on therapeutic decisions in routine clinical practice due to the absence of novel drugs. Then, outside of clinical trials, only younger “fit” patients underwent cytogenetic analysis as part of the qualification process for standard/tandem AHSCT. For example, in a recent study of the Polish Myeloma Group in 197 patients with early mortality, the cytogenetics data were available for only 59 patients (71). Only recent reimbursement of novel drugs in Poland, including daratumumab (2019), carfilzomib (2019), and ixazomib (2021), has led to routine cytogenetics testing. To compensate for missing cytogenetics data, in the final model predicting CR after AHSCT we included only one clinical variable- response after induction therapy. This model achieved satisfactory accuracy in predicting CR achievement after AHSCT. We hypothesize that adding cytogenetics data may potentially increase the clinical value of our observations, but we cannot address this issue because of the lack of collected bone marrow samples for conducting FISH/cytogenetics.

hsa-miR-223-3p could serve as a prognostic indicator, assisting in identifying patients likely to have favorable long-term outcomes. Importantly, our study adds to the growing body of literature by providing evidence for the predictive value of miRNA signatures, specifically in AHSCT. By evaluating miRNA expression profiles during the AHSCT procedure, our findings offer unique insights into the predictive role of miRNAs in this setting, which may have translational relevance for posttransplant treatment decisions. Further validation of our miRNA signature in larger cohorts and prospective studies may lead to the development of clinically useful prognostic biomarkers to guide treatment decisions and improve outcomes in MM patients undergoing AHSCT.

## Data availability statement

The original contributions presented in the study are included in the article/**Supplementary Material**, further inquiries can be directed to the corresponding author/s.

## Ethics statement

The studies involving humans were approved by The Ethical Committee of the Medical University of Lodz (No RNN/424/19/KE). The studies were conducted in accordance with the local legislation and institutional requirements. The participants provided their written informed consent to participate in this study.

## Author contributions

DM wrote the first version of the manuscript. DM and WF designed and planned the experiments. DM, MN, MM, KPK, KO, KiK collected the data. DM, MN, MM and KiK collected the samples. DM and IZ performed the experiments. DM, KPK, KO and WF performed statistical analyses. All authors reviewed and approved the submitted version. All authors contributed to the article.

## Supplementary material

Supplementary Table 1 Expression of selected miRNAs according to the ISS stage.

Supplementary File 1 Normalized miRNA expression data with clinical variables.

Supplementary File 2 Weka dataset for classifier construction and 10-fold cross-validation.

Supplementary File 3 Final RandomTree classifier file.
