# Supplementary material for: High serum miR-223-3p expression level predicts complete response and prolonged overall survival in multiple myeloma patients undergoing autologous hematopoietic stem cell transplantation
Source: Front Oncol. 2023 Sep 27;13:1250355. doi: 10.3389/fonc.2023.1250355 (PMC10565214; doi:10.3389/fonc.2023.1250355)
Supplement: Supplementary Table 1 — Expression of selected miRNAs according to the ISS stage. [file Table_1.docx]

**Supplementary Table 1**. Expression of selected miRNAs according to the ISS stage.

| miRNA | ISS I/II  Mean ΔCt | ISS I/II  SD | ISS III  Mean  ΔCt | ISS III  SD | FC | log2FC | p-value |
| --- | --- | --- | --- | --- | --- | --- | --- |
| hsa-miR-223-3p T1 | 4.78 | 0.61 | 4.37 | 0.46 | 0.76 | -0.40 | 0.0155 |
| hsa-miR-320c T1 | 0.26 | 0.57 | 0.60 | 0.55 | 1.27 | 0.35 | 0.0470 |
| hsa-miR-221-3p T1 | 0.52 | 0.46 | 0.34 | 0.56 | 0.88 | -0.18 | 0.2563 |
| hsa-miR-15b-5p T1 | 0.08 | 0.73 | 0.34 | 0.82 | 1.20 | 0.26 | 0.2828 |
| hsa-miR-150-5p T1 | -0.45 | 0.73 | -0.27 | 0.91 | 1.14 | 0.19 | 0.4597 |
| hsa-miR-221-3p T1 | 0.03 | 0.52 | 0.16 | 0.60 | 1.10 | 0.13 | 0.4448 |
| hsa-miR-320c T2 | 0.79 | 0.77 | 1.00 | 0.88 | 1.15 | 0.21 | 0.4195 |
| hsa-miR-361-3p T2 | -5.23 | 0.57 | -5.18 | 0.58 | 1.04 | 0.05 | 0.7659 |
| hsa-miR-361-3p T2 | -4.85 | 1.07 | -4.71 | 1.13 | 1.10 | 0.14 | 0.6717 |
| hsa-miR-150-5p T2 | -0.71 | 0.66 | -0.77 | 0.89 | 0.96 | -0.06 | 0.8010 |
| hsa-miR-15b-5p T2 | -0.73 | 0.68 | -0.72 | 0.81 | 1.01 | 0.01 | 0.9719 |
| hsa-miR-223-3p T2 | 4.24 | 0.88 | 4.21 | 0.99 | 0.98 | -0.03 | 0.9249 |
